# The Nerves of Taste

**Published:** 1872-09

**Authors:** 


### The Nerves of Taste.

A fresh contribution to our knowledge of this subject has been given by Dr. Ph. Lussana, in the last number of the *Archives de Physiologie*; his views being based on pathology. He gives the details of an Italian peasant woman, from whom Dr. Vanzetti twice excised a portion of the lingual nerve; two years afterward it was found that the tongue was normal in form, nutrition, and color on the affected side, but had entirely lost its gustatory sensibility, sweets and bitters being alike unperceived. This result he points out might have qeen anticipated by our knowledge of anatomy, since it is known that the lingual alone ramifies in the mucous membrane of the anterior part of the tongue to the exclusion of the glosso pharyngeal, which is distributed to the posterior region of the lingual mucous membrane alone. He next proceeds to show that the gustatory fibres of the lingual nerve *do not come from the fifth*, and this he proves by adducing cases of complete paralyses of the fifth, yet in which taste was perfectly preserved. They come from the seventh, as is shown by the loss of taste when the facial was divided or paralyzed, and are, in fact, contained in the chorda tympani.—*London Lancet.*

